# Nonsurgical management of a large periapical lesion using aspiration in combination with a triple antibiotic paste and calcium hydroxide

**Published:** 2010-11-15

**Authors:** Marina Fernandes

**Affiliations:** Goa Dental College and Hospital, India

**Keywords:** Periapical Diseases, Root Canal Medicaments, Antibiotics, Calcium Hydroxide, Retreatment

## Abstract

Persistent microorganisms in the root canal are known to cause endodontic treatments failure. Overextended gutta-percha can also act as a periradicular tissue irritant, leading to large periapical lesions. Retrieval of overextended gutta-percha with a nonsurgical approach can prove to be a challenge. This case describes the nonsurgical management of a large periapical lesion associated with overextended gutta-percha. Retrieval of gutta-percha was attempted but the overextended portion could not be removed. Aspiration of the purulent exudate was done through the root canal followed by use of a triple antibiotic paste. After 2 weeks the antibiotic paste was replaced with calcium hydroxide, to enhance the osseous regeneration. The periapical lesion showed a considerable amount of periapical healing after 15 months. The results of this case demonstrate that aspiration in conjunction with the triple antibiotic paste and calcium hydroxide may possibly be used in managing large periapical lesions associated with overextend gutta-percha.

## Introduction

The main reason for root canal treatment failure is the persistent microorganisms that remain after therapy or recontamination of the canal system because of an inadequate seal (1). Gutta-percha, the most commonly used root filling material, can also act as a periradicular tissue irritant, if it extends beyond the apical foramen (2,3). Large periapical lesions can be associated with such teeth. There is clinical evidence that as the periapical lesion increase in size, the incidence of radicular cysts increases. However, lesions with ≥20mm diameters have been shown to be granulomas (4). 

Treatment options to manage large periapical lesions range from nonsurgical root canal treatment and apical surgery to extraction (5). However, a growing interest in endodontic retreatment has occurred stemming from patients’ increased demand to preserve teeth (1). Adopting a surgical approach requires consideration of several key factors such as proximity of the lesion to adjacent vital teeth, encroachment on anatomical structures, patient cooperation, and treatment duration (4,5). 

Various nonsurgical methods have been used in the management of periapical lesions (5-11). When a nonsurgical retreatment approach is adopted in a tooth with overextended gutta-percha, retrieval of the overextended gutta-percha can prove to be challenging. 

The following case reports the management of a large periapical lesion associated with overextended gutta-percha, using aspiration in combination with a triple antibiotic paste and calcium hydroxide, as the surgical approach could not be adopted due to patient refusal.

**Figure 1:**
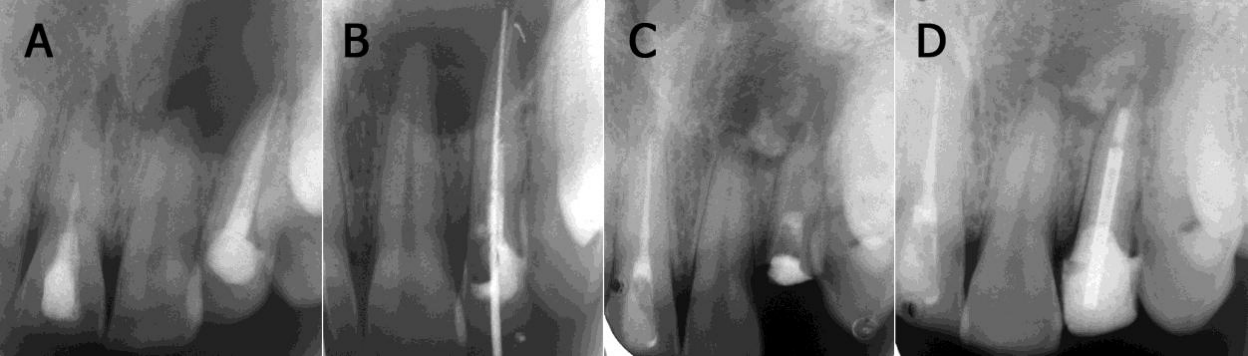
A) Periapical radiograph showing a large periapical radiolucency apparently involving the apices of teeth #9 and 10. Tooth #10 shows a radiopaque material extending beyond the radiographic apex by approximately 2.5mm. B) Periapical radiograph showing gutta-percha beyond the root apex which could not be retrieved and a no. 40 hand K-file that passed beyond the apical foramen. C) Periapical radiograph after 5 months showing a favorable amount of periapical healing. The weakened coronal tooth structure was removed. D) Periapical radiograph after 1 year 3 months showing considerable amount of periapical healing in spite of the overextended gutta-percha

## Case Report

A 43-year-old male patient was referred to the department of Conservative Dentistry and Endodontics at Goa Dental College Hospital, with pain and palatal swelling in the maxillary left anterior region. Patient gave a history of trauma to his anterior teeth about 2 years ago, which had been treated by a general dentist. 

One year post operatively, the patient developed a palatal swelling; the patient took self-administered antibiotics and analgesics during this one year period, however the swelling repeatedly recurred.

Intraoral examination revealed a firm palatal swelling extending from tooth #9 to 11. Teeth #8 and 10 were discoloured and failed to respond to pulp sensitivity testing. The adjacent teeth responded within normal limits.

A periapical radiograph revealed a large periapical radiolucency (~1.7×1.4cm in diameter) apparently involving the apices of teeth #9 and 10 (Figure 1A). Tooth #10 showed a radiopaque material that extended from the root canal space and beyond the radiographic apex by approximately 2.5mm. Inadequate adaptation of the gutta-percha at the apex was also noted which probably further exacerbated the poor apical seal. Tooth #8 showed a radiopaque material extending only till the middle third of the root canal space. A radiolucent bulge in middle third of the root canal was suggestive of an internal resorptive defect.

A surgical approach was offered for treating the periapical lesion, however the patient refused it. Hence, it was decided to adopt a nonsurgical retreatment approach for tooth #10. It was also decided to simultaneously retreat tooth #8 keeping in mind the poor quality of obturation and the possibility of internal resorption.

Following removal of the palatal restoration from tooth #10, gutta-percha obturating material was seen at the root canal orifice. A weakened coronal tooth structure was also noted which would warrant a post placement following the endodontic treatment. Removal of the gutta-percha was then attempted. Space was created between the gutta-percha and the canal wall using hand K-files (Dentsply Maillefer, Ballaigues, Switzerland) and the gutta-percha was retrieved from the root canal using hand H-files (Dentsply Maillefer, Ballaigues, Switzerland). No solvent was used for the purpose. However, the gutta-percha beyond the root apex could not be retrieved. A hand K-file size #40 freely passed beyond the apical foramen (Figure 1B). Digital pressure was applied on the palatal swelling which expressed purulent exudate from the canal. The tooth was instrumented to ISO size 50 by using hand K-files with 3% sodium hypochlorite (Vensons India, Bangalore, India) as irrigation between instruments. The purulent exudate was aspirated as described by Fernandes and Ataide by passing a 25 gauge needle attached to a 5mL syringe beyond the apical foramen (11). Finger pressure was applied on the swelling while the canal was dried with paper points (Dentsply Maillefer, Ballaigues, Switzerland) and subsequently temporized. At the end of the appointment the palatal swelling had decreased in size. However, at the next appointment after one week post operatively, the patient complained of pain and similar palatal swelling; the temporary restoration was also lost and there was purulent discharge from the canal. It was then decided to use the triple antibiotic paste of ciprofloxacin 500mg (Ciplox 500mg, Cipla Ltd, Sikkim, India), metronidazole 400mg (Aristogyl, Aristo Pharmaceuticals Pvt Ltd, India) and minocycline 100mg (Ranbaxy Laboratories Ltd, Solan, India). Patient was not allergic to the antibiotics used. A creamy paste as described by Takushige *et al. *was prepared (12). Following aspiration of the purulent exudate and drying of the canal, the paste was used as an intracanal medicament. Retreatment of tooth #8 was also initiated and completed at the following appointments. After 2 weeks, the patient was asymptomatic and there was no evidence of any swelling. The triple antibiotic paste was irrigated out of the canal with 3% sodium hypochlorite. There was no purulent discharge from the canal. The canal was dried and calcium hydroxide paste (RC Cal, Prime Dental, India) was used as an intracanal medicament allowing some extrusion in the periapical area. The calcium hydroxide was then exchanged every month. After 5 months, a favourable amount of periapical healing was observed (Figure 1C). The weakened coronal tooth structure was removed and the root canal was obturated, followed by post placement and core build up. A radiograph taken after 15 months showed considerable periapical healing (Figure 1D). The patient was asymptomatic and scheduled for another follow up appointment.

## Discussion

Drainage is important in the conservative management of large periapical lesions. When direct and immediate drainage is obtained from localized swellings, abscesses or cysts, the symptoms are reduced and fibroplasia and cell migration can occur (13,14). Fernandes and Ataide had reported use of a simple aspiration through the root canal in management of a large periapical lesion (11). Aspiration of an unknown bony lesion is a relatively innocuous surgical procedure which may provide information regarding the presence of purulence, cystic fluid or haemorrhage. The purulent nature of the exudate in the present case could explain the inadequacy of the aspiration technique alone. This fact was also reported earlier by Hoen *et al.* who described the aspiration and irrigation technique using the buccal-palatal approach (7). They stated that the technique could be used only in lesions that were apparently uninfected periapical cysts. This conclusion was based on the type of fluid removed and the lack of microbial presence in both culture and gram stain specimens. 

The triple antibiotic paste has been proven to be effective in eliminating bacteria from infected dental tissues (12,15,16). Metronidazole has a wide bactericidal spectrum against anaerobes. Ciprofloxacin and minocycline are effective against bacteria resistant to metronidazole (16). Despite the little amount of used medicament, sensitivity of patients to chemicals or antibiotics should be ruled out before their use (17). The disadvantage of the triple antibiotic paste is tooth discoloration induced by minocycline. Cefaclor and fosfomycin are proposed as alternatives to minocycline in terms of their antibiotic effectiveness, but further clinical studies are needed to demonstrate their effectiveness in the root canal (18). In the present case report, the antibiotic paste was used due to the purulent nature of the exudate for a period of 2 weeks. It was then replaced with calcium hydroxide to enhance the osseous regeneration of the osseous cavity. Many investigators advocate that direct contact between the calcium hydroxide and the periapical tissues is beneficial for the Osseo-inductive property of the material (19,20). A high degree of success has been reported by using calcium hydroxide beyond the apex in cases with large periapical lesions (21). Hence calcium hydroxide was intentionally extruded beyond the apical foramen, which resulted in favourable osseous regeneration in spite of the overextended gutta-percha. 

Patient cooperation is an important factor when using a surgical approach in managing periapical lesions. Using aspiration along with the triple antibiotic paste and calcium hydroxide can aid in management of nonsurgical cases. 

## Conclusion

Failed endodontic therapies can be good candidates for nonsurgical treatment. Thorough cleaning and shaping of the canal along with disinfection are key factors in initiating periapical healing. Various treatment modalities like the use of the triple antibiotic paste, and calcium hydroxide can enhance and accelerate the healing process.
